# Progress and Prospects of the Molecular Basis of Soybean Cold Tolerance

**DOI:** 10.3390/plants12030459

**Published:** 2023-01-19

**Authors:** Mesfin Tsegaw, Workie Anley Zegeye, Bingjun Jiang, Shi Sun, Shan Yuan, Tianfu Han, Tingting Wu

**Affiliations:** 1 MARA Key Laboratory of Soybean Biology (Beijing), Institute of Crop Sciences, Chinese Academy of Agricultural Sciences, Beijing 100081, China; 2 Department of Agricultural Biotechnology, Institute of Biotechnology, University of Gondar, Gondar P.O. Box 194, Ethiopia; 3 John Innes Centre, Norwich Bioscience Institutes, Norwich NR2 3LA, UK

**Keywords:** soybean, cold stress, CBF, molecular pathways, transcription factors

## Abstract

Cold stress is a major factor influencing the geographical distribution of soybean growth and causes immense losses in productivity. Understanding the molecular mechanisms that the soybean has undergone to survive cold temperatures will have immense value in improving soybean cold tolerance. This review focuses on the molecular mechanisms involved in soybean response to cold. We summarized the recent studies on soybean cold-tolerant quantitative trait loci (QTLs), transcription factors, associated cold-regulated (*COR*) genes, and the regulatory pathways in response to cold stress. Cold-tolerant QTLs were found to be overlapped with the genomic region of maturity loci of *E1*, *E3*, *E4*, pubescence color locus of *T*, stem growth habit gene locus of *Dt1*, and leaf shape locus of *Ln*, indicating that pleiotropic loci may control multiple traits, including cold tolerance. The C-repeat responsive element binding factors (CBFs) are evolutionarily conserved across species. The expression of most *GmDREB1s* was upregulated by cold stress and overexpression of *GmDREB1B;1* in soybean protoplast, and transgenic Arabidopsis plants can increase the expression of genes with the DRE core motif in their promoter regions under cold stress. Other soybean cold-responsive regulators, such as *GmMYBJ1*, *GmNEK1*, *GmZF1*, *GmbZIP*, *GmTCF1a, SCOF-1* and so on, enhance cold tolerance by regulating the expression of *COR* genes in transgenic Arabidopsis. CBF-dependent and CBF-independent pathways are cross-talking and work together to activate cold stress gene expression. Even though it requires further dissection for precise understanding, the function of soybean cold-responsive transcription factors and associated *COR* genes studied in Arabidopsis shed light on the molecular mechanism of cold responses in soybeans and other crops. Furthermore, the findings may also provide practical applications for breeding cold-tolerant soybean varieties in high-latitude and high-altitude regions.

## 1. Introduction

The soybean (*Glycine max* (L.) Merr.) is an important food crop and a valuable source of protein and oil in human and animal diets worldwide [1]. It fixes natural nitrogen by nodulation in the roots which improves soil fertility [2]. The soybean has been grown worldwide, including in high-latitude and high-altitude regions with cold temperatures [3]. Soybean cold-production regions are mainly distributed in China (the regions located between N 47° and N 53°34′), Russia (the far-eastern region, particularly in Amurskaya Oblast), and Canada (Ontario, Quebec, and Manitoba) [4,5] (http://www.soybeancouncil.ca, accessed on 20 January 2022). Because of the cold temperatures in these regions, a lack of cold tolerance imposes prominent negative influences on soybean nodulation, yield, and seed quality [6].

It is widely agreed that cold stress is one of the major environmental factors that limit agricultural productivity because it retards plant development and growth, which has a huge impact on the adaptation and distribution of plants [7,8]. Cold includes chilling (0–15 °C) and freezing (<0 °C) temperatures. The difference between chilling and freezing stress is that chilling stress induces rigidified membranes, destabilized proteins, and damaged photosynthetic apparatus. In contrast, freezing stress causes more severe injuries due to ice formation and cell dehydration [9,10,11]. The irregular appearance of chilling temperatures is the common environmental stress affecting the growth and development of soybean grown at high latitudes and high altitudes. This temperature is non-freezing but cool enough to produce soybean injury [12]. There are two types of chilling injuries: primary and secondary. The primary chilling injury is instant and reversible, whereas the secondary chilling injury is irreversible when the plant is exposed to a non-chilling temperature [13]. Plants can use different strategies and undergo physiological reactions to adapt to chilling-temperature conditions. For example, concentrations of endogenous ABA and ascorbic acid were increased in the leaves and shoots in response to the chilling temperature. The increase in cold tolerance after exposure to a short period of chilling temperature is referred to as cold acclimation [14,15]. The unusual climate experienced recently increases the importance of breeding crops tolerant to chilling temperatures. Therefore, one way to improve crop cultivation in the chilling temperature regions is by understanding the molecular basis of cold stress responses. Identification of genetic resources and genes that confer chilling tolerance is also a major interest among soybean breeders in cold regions. Throughout this review, we demonstrate the headway that has been made and the advancement that has been achieved in understanding the molecular mechanism underlying chilling response and acclimation in soybeans.

## 2. Influence of Cold Stress on the Growth and Development of Soybeans

As a major environmental factor, cold stress limits where crops can be grown and reduces yields. Cold stress reduced soybean seed yield on average by 24% compared to unaffected plants [16]. Crops like soybeans, which are native to temperate areas, need warm temperatures for germination, growth, development, and maturation. The optimum temperatures for soybean germination, flowering, and maturity are 15–22 °C, 20–25 °C, and 15–22 °C, respectively [17]. They exhibit symptoms of injury when exposed to low non-freezing temperatures. The chilling temperature affects soybean development from germination to maturity. Soybean seeds are sensitive to the chilling temperature during germination [18]. When the average air temperature drops below 15 °C, it causes growth retardation and the inhibition of new leaf and shoot production in soybean, while a drop below 10 °C may even cause them to fail to flower [19]. Chilling temperature stress during flowering has led to decreased pollen density and, consequently, decreased pod setting and a remarkable reduction in seed yield [20,21,22]. Gass and Schori [23] also reported irregular distribution of pods and seeds along the stem due to cold stress in the soybean. Inhibition of pod formation occurred when the minimum temperature of a single night dropped to 8 °C [24]. In the winter nursery of Hainan, aborted or infertile pods are common when chilling weather occurs. Cold stress prevents soybean growth by inhibiting metabolic and physiological activities, such as water uptake, cellular dehydration, and oxidative stresses [25,26]. Aquaphotomics analysis revealed major changes in the water molecular structure in soybean leaves, as well as altered carbohydrate and oxidative metabolism in response to cold stress [27].

## 3. Candidate Genes/QTLs Associated with Cold Tolerance in Soybean

The exploitation of stress-tolerant genes and quantitative trait loci (QTLs) are effective ways to generate stress-tolerant crops [8]. Chen et al. [28] have identified 422 SNPs and 302 genes associated with drought tolerance by using 136 soybean genotypes under well-watered and drought conditions. The study confirmed that these important loci and potential genes are valuable for soybean drought-tolerance breeding programs. Soybean breeders and researchers have also identified several QTLs associated with chilling tolerance (Table 1). Among them, the locus *T/t* (tawny/gray pubescence) has been studied most intensively. Cultivars and lines with the T allele at the *T* locus, which controls the color of pubescence, have repeatedly been demonstrated to exhibit better chilling tolerance than those with the alternative allele, *t* [20,21,29]. The pod settings in soybeans under short-term and long-term cold treatments were different across cultivars. An earlier study reported a strong relationship between one of the soybean genes regulated by cold (*Src2*) transcript accumulation and chilling tolerance in soybean seedlings [12]. However, evidence showing that these loci are directly involved in chilling tolerance remains to be provided. There is also a genetic association between ascorbate peroxidase (APX1) isozyme, a ROS-scavenging enzyme, and chilling tolerance in soybeans, which shows that APX1 deficiency boosts tolerance against the chilling temperature. Although it is indicated that there is no linkage between the *apx1* locus and the previously reported loci associated with chilling tolerance, further experiments may be needed to verify its linkage relationship with recently identified QTLs [30]. Maturity loci known to control maturity such as *E1*, *E3,* and *E4* were also found to be associated with chilling tolerance for both seed yield and quality [31]. The *E1* allele was found to be more important than the *e1* allele in chilling tolerance [32]. Moreover, three QTLs, *qCTTSW1*, *qCTTSW2,* and *qCTTSW3*, were detected for chilling tolerance in seed-yielding ability. The two QTLs, *qCTTSW1* and *qCTTSW2*, were mapped near the QTLs for flowering time [31]. Stem growth habit gene (*Dt1*), regulators of leaf shape (*Ln*), and pubescence density (*P1*) loci have been associated with chilling tolerance in soybeans [33]. Out of the five QTLs for pigmentation associated with chilling, identified by Githiri et al. [34], two of them, *fd2* and *fd4*, are believed to correspond to maturity genes *E1* and *E3*, respectively.

Twelve QTLs were detected for tolerance to chilling temperature during germination by one-way ANOVA. More than 28 QTLs related to cold acclimatization were identified from genome-wide studies in soybeans during germination and seedling stages (Table 1). Of these, 10 QTLs were detected at both stages, indicating the possibility of developing a soybean variety of chilling-temperature tolerance for both stages using these kinds of overlapping QTLs [8]. A QTL analysis around soybean inverted-repeat CHS pseudogene (*GmIRCHS)* showed that *GmIRCHS* or a region located very close to it was responsible for cold tolerance [35].

**Table 1:** List of genes, QTLs, and molecular markers associated with soybean cold tolerance and their genomic location.

Candidate Genes/QTLs/Associated Markers	Chromosome/Linkage Group	References
*APXI*	11(B1)	[30]
*T*	6(C2)	[31,32,36,37,38]
*E1*	6(C2)	[32,38]
*E4*	20(I)	[31,38]
*Ln*	20(I)	[33]
*PI*	9(K)	[33]
*Dt1*	19(L)	[33]
*E3*	19(L)	[31,38]
*qCTTSW1*	6(C2)	[31]
*qCTTSW2*	19(L)	[31]
*qCTTSW3*	12(H)	[31]
*GmIRCHS*	8(A2)	[35]
Sat_342	14(B2)	[35]
*qCS8-1*	8(A2)	[39]
*qCS11-1*	8(B1)	[39]
*Ic*	8(A2)	[35,40]
*S07_42231812*	7(M)	[41]
*S11_30574868*	11(B1)	[41]
*S13_33041524*	13(F)	[41]
Sat_271	5(A1)	[8]
Satt225	5(A1)	
Sat_331	11(B1)	
Satt168	14(B2)	
Satt577	14(B2)	
Satt338	4(C1)	
Satt640	6(C2)	
Satt041	1(D1b)	
Satt271	1(D1b)	
Satt458	17(D2)	
Satt669	17(D2)	
Satt651	15(E)	
Satt142	12(H)	
Satt253	12(H)	
Satt353	12(H)	
Satt440	20(I)	
Satt249	16(J)	
Sat_126	9(K)	
Satt240	9(K)	
Satt349	9(K)	
Satt513	19(L)	
Sat_244	7(M)	
Satt323	7(M)	
Satt336	7(M)	
Satt540	7(M)	
Sat_192	1(D1b)	
Satt663	13(F)	
Sat_020	9(K)	

Additionally, some of the QTLs, listed in Table 1, are indirectly related to cold response, for instance, with either seed coat discoloration, seed coat cracking, or pod number under cold stresses. Yamaguchi et al. [39] identified QTLs, *qCS8-1,* and *qCS11-1*, for seed coat cracking tolerance under chilling stress at the reproductive stage. The *qCS8-1* and *qCS11-1* loci were found to have no negative influence on flowering time or other agronomic traits. Recently, Yamaguchi et al. [40] identified QTLs located in the proximal region of the *I* locus associated with cold-induced seed coat discoloration (CD). Likewise, Jähne et al. [41] also identified cold temperature-specific QTLs (S07_42231812, S11_30574868, and S13_33041524) for pod numbers under stressed conditions of cold temperature on chromosomes 7, 11, and 13, respectively. The cold-specific QTLs identified on chromosome 11 by this group required further confirmation to test whether it is identical to the seed coat cracking QTL (*qCS11-1*) identified by Yamaguchi et al. [39].

## 4. Soybean Cold Responsive Regulators and Associated Cold Regulated (*COR)* Genes

When plants are exposed to cold stress, there will be a perception of a cold signal and a rise in Ca^2+^, plant hormones, and reactive oxygen species (ROS) which triggers the expression of key transcription factors like CBF and other regulators. In turn, CBF and other regulators activate the expression of downstream *COR* genes, such as *RD29A*, *RD17*, *COR6.6*, *COR15A*, *ERD10*, *KIN1*, *KIN2,* and others in response to cold [42,43,44,45]. Some of these genes encode key enzymes for osmolyte biosynthesis that can induce the increase of cryoprotective proteins and soluble sugars, repair cold-rigidified membranes, and stabilize osmotic potential [25]. While there are a lot of studies conducted on the phenotypic response of soybeans to chilling temperatures, only a limited number of cold regulators have been studied to understand the genetic mechanism in soybean cold-tolerance including GmDREB3 [46,47], GmDREB1 [48], GmDREB1A;2 and GmDREB1B;1 [49], soybean zinc finger protein, SCOF-1 [50], GmEIN3 [51], KS dehydrin [52], and soybean NIMA-Related Kinase1 [53]. Thus, understanding the genetic mechanism in soybean response to chilling temperature will pave the way for the development of cold-tolerant genotypes.

### 4.1. Soybean CBF-Dependent Cold Response Regulatory Pathway

The C-repeat binding factor/dehydration-responsive element binding factor (CBF/DREB) belonging to AP2/ERF family transcription factors plays important roles in cold temperature response in plants [54]. The CBF/DREB1 transcription factor can bind to the promoter’s CRT/DRE (C-repeat/dehydration-responsive element) region in cold-responsive genes [55]. *AtCBFs* can function as transcriptional activators that bind to the CRT/DRE regulatory element of cold-regulated genes in Arabidopsis [56]. It has been found that *CBF* genes are conserved in plants that can or cannot acclimate to cold temperatures, including rice (*Oryza sativa*), tomato (*Solanum lycopersicum*), rapeseed (*Brassica napus*), wheat (*Triticum aestivum*), barley (*Hordeum vulgare*), maize (*Zea mays*), and soybean (*Glycine max*) [56,57,58]. A total of 44 homologs of Arabidopsis CBFs have been identified from soybeans [49]. Unlike the tandem array arrangement of three Arabidopsis CBFs in the same chromosome, soybean CBF genes were duplicated and scattered among different chromosomes [49].

The expression of most *GmDREB1s* was upregulated by abiotic stresses, such as cold, salt, drought and heat stresses [48]. Seven *GmDREB1s*, *GmDREB1A;1*, *GmDREB1A;2*, *GmDREB1B;1*, *GmDREB1B;2*, *GmDREB1C;1*, *GmDREB1D;1*, and *GmDREB1D;2* were significantly upregulated after an hour and remained elevated at 24 h under cold stress [48,49]. Another soybean *DREB* gene, *GmDREB3*, was upregulated after 0.5 h of cold treatment and was not detected in 3 h after cold treatment. Overexpression of *GmDREB3* improved drought, high salt, and cold stress tolerance in transgenic Arabidopsis [46]. Overexpression of *GmDREB1B;1* in soybean protoplast can increase the expression of genes with the DRE core motif in their promoter regions under abiotic stress conditions. *GmDREB1B;1* can activate many soybean-specific cold-responsive genes including *GmPYL21*, an ABA receptor family gene, which could activate the downstream ABRE-mediated gene expression in an ABA-independent way. Soybean *DREB1s* were demonstrated to upregulate the transcription of downstream *COR* genes *AtCOR47* and *AtRD29a* and increase cold tolerance in transgenic Arabidopsis [48]. Regulated by *DREB1* genes, *GmVRN1* is strongly accumulated in the *AtDREB1A*-overexpressing soybean [59]. *GmVRN1* is responsive to low temperatures and is believed to participate in the vernalization pathway in transgenic Arabidopsis to regulate flowering time. This feature of *GmVRN1* in soybeans is different from its role in transgenic Arabidopsis [60]. Moreover, overexpression of *VRN1*, the homolog of *GmVRN1*, causes early flowering in Arabidopsis [61]. *AtDREB1A* could bind to the DRE motif, which is 157 to 186 bp upstream of *GmVRN1*, indicating that the effect of *GmVRN1* may be mediated by a CBF-dependent cold-responsive pathway [60]. Further dissection of the molecular crosstalk between the cold response pathway and flower regulation by *GmVRN1* will help uncover its role in downstream *COR* gene regulation.

The soybean ethylene pathway is found to regulate the soybean CBF/DREB1 cold-responsive pathway by the accumulation of transcripts encoding the transcription factor GmEIN3 in response to the cold [51]. GmEIN3 inhibits the CBF/DREB1 pathway in soybeans. Transcription of soybean *DREB1A;1* and *DREB1B;1* was found to be upregulated by the cold, and their levels were consistently increased and decreased by ethylene pathway inhibitors and stimulators, respectively. Further studies would be required to determine whether *GmEIN3* negatively regulates *GmDREB1A;1* by binding to the DRE motif in the *GmDREB1A;1* promoter during cold stress.

Besides the CBF-dependent (DRE-mediated) pathway, the CBF-independent pathway, which is activated by other transcription factors rather than CBF/DREB, such as MYB, bZIP, WRKY, and Zinc Finger-type transcription factors [50,62,63,64], is also important for soybean cold-tolerance. Transcription factors in the CBF-independent pathway mostly have a cis-acting element named the ABA-responsive element (ABRE; ACGTGG/TC) in their promoter regions. The ABA-responsive element (ABRE) controls the transcription of downstream target genes through the ABRE-binding protein/ABRE-binding factor (AREB/ABF). Contrary to the belief that DRE-mediated (CBF-dependent) and ABRE-mediated (CBF-independent) pathways are known to act in a parallel manner, the findings reveal that these pathways cross-talk and work together to activate cold stress gene expression in Arabidopsis [65]. Narusaka et al. [66] also reported that DRE-mediated and ABRE-mediated gene expressions are interdependent in the stress-responsive expression of *COR* genes like *RD29A*. Similarly, *GmDREB1B;1* directly activates *GmPYL21* expression and enhances ABRE-mediated gene expression in soybeans [48].

### 4.2. Other Soybean Cold Response Regulatory Pathways

Several soybean genes have been shown to mediate cold tolerance (Table 2). Soybean cold-responsive genes function in multiple signaling pathways in transgenic Arabidopsis in response to chilling temperature stress in addition to the CBF-dependent pathway. Over-expression of soybean regulators, such as *GmMYBJ1* (R2R3-type *MYB* genes), *GmZF1* and *SCOF-1* (C2H2 zinc finger gene), *GmbZIP44*, *GmbZIP62* and *GmbZIP78* (bZIP transcriptional factors), *GmTCF1a* (regulator of chromosome condensation 1 (RCC1) family genes), *GmNEK1* (NEK family gene), and *GmWRKY21* (WRKY-type transcription factor) in Arabidopsis can enhance cold tolerance [50,53,62,63,64,67,68]. Most of them enhance the expression of downstream *COR* genes in a CBF-independent pathway. Different regulators induce different target *COR* genes in transgenic Arabidopsis, e.g., *GmMYBJ1 (AtRD29b*, *AtCOR47*, *AtCOR78,* and *AtCOR15a)*, *GmZF1 (AtCOR6.6)*, *GmbZIPs* (*AtERF5*, *AtKIN1*, *AtCOR78, and AtCOR15a)*, *GmTCF1a (AtCOR15a),* and *SCOF-1 (AtCOR15a*, *AtRD29B*, and *AtCOR47*).

In a CBF-independent pathway, there is a slight downregulation in the expression of *AtDREB2A* in the *GmMYBJ1* transgenic Arabidopsis, suggesting the expression of stress-responsive *COR* genes may be regulated independently of CBFs [62]. A high expression level of *GmZF1* mRNA induced by exogenous ABA suggested that *GmZF1* was also involved in a CBF-independent signal transduction pathway in transgenic Arabidopsis [67]. The ectopic expression of *GmTCF1a* does not alter the expression of *CBFs* in Arabidopsis, suggesting that the impact of *GmTCF1a* in overexpressing Arabidopsis is independent of the CBF pathway [68]. Its ortholog from Arabidopsis, *AtTCF1*, regulates freezing tolerance through a CBF-independent pathway [69].

**Figure 1:**
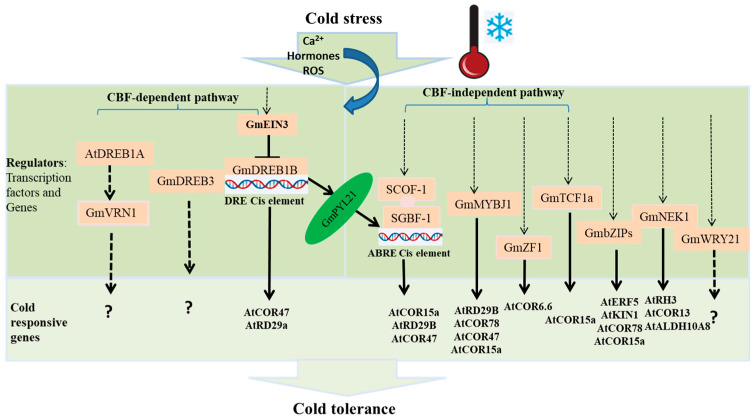
Soybean transcription factors and genes responsive to cold stress. In the CBF-dependent pathway, *GmEIN3* inhibits the GmDREB1B pathway in soybeans, and soybean *GmDREB1s* up-regulate the transcription of downstream *COR* genes in transgenic Arabidopsis. *GmVRN1* is found to be upregulated in *AtDREB1B:1*-overexpressing soybeans. In the CBF-independent pathway, *GmMYBJ1*, *GmZF1,* and *GmTCF1a* upregulate one or more *COR* genes in transgenic Arabidopsis. *SCOF-1* specifically enhanced SGBF-1 binding to ABRE and activated ABRE-mediated *COR* gene expression in transgenic Arabidopsis. Whether *GmWRKY*, *GmbZIPs*, and *GmNEK1* are in CBF-dependent pathways remains to be studied. DRE-mediated and ABRE-mediated gene expressions are interdependent. *GmDREB1B;1* directly activates *GmPYL21* expression, and *GmPYL21* enhances ABRE-mediated gene expression in soybeans. Dotted lines indicate signaling pathways that remain unclear. Arrows indicate positive regulation and T-bars indicate negative regulation.

*SCOF-1*, a novel cold regulator specific to soybeans, functions as a positive regulator of *COR* gene expression to enhance cold tolerance in transgenic Arabidopsis and tobacco [50]. Unlike the above common chilling response regulators, *SCOF-1* did not bind directly to CRT/DRE or ABRE in the promoter of *COR* genes; instead, it greatly enhanced the DNA binding activity of a soybean G-box binding bZIP transcription factor, *SGBF-1* [50]. Hence, the binding enhancement of *SGBF-1* by *SCOF-1* is *CBF*-independent in transgenic Arabidopsis. Soybean *SCOF-1* has a considerable sequence similarity with Arabidopsis *AZF* and *STZ*, which act as negative regulators of *COR* gene expression in Arabidopsis [70]. Therefore, further research can be conducted to ascertain whether *SCOF1*-like proteins are negative or positive regulators of soybean COR genes following cold treatments. No data was provided on the *COR* genes regulated by *GmWRKY21* (Figure 1). *CsWRKY46* in cucumber and *WKRY6* in Arabidopsis were found to participate in cold response in the non-CBF module [71]. However, whether *GmWRKY* interacts with *CBF* and the DRE-cis-acting promoter region is yet to be explored.

Concerning soybeans’ ability for cold acclimation, an acid dehydrin family member *COR* gene, *GmERD14*, was identified from soybeans and characterized for its response to coldstress. There was little to no cold-responsive accumulation of dehydrins in soybean compared to that in cold-tolerant and cold-acclimating plants [52]. The lack of cold stress-regulated acidic dehydrin expression may contribute to the mildly cold acclimation of soybeans. At the protein level, chilling acclimation-related proteins were characterized based on the protein synthesis profile during soybean chilling treatment. Molecular characterization of the protein associated with chilling adaptation indicated that one of the members in the heat shock protein 70 (HSP70) family may enhance the capacity of soybeans’ chilling acclimation [72].

**Table 2:** Regulators identified from soybeans exhibited function related to cold stress as indicated in Arabidopsis and soybeans.

Factor	Gene Characteristics	Effect on Cold Tolerance	References
*GmDREB1s*	*DREB* genes	Arabidopsis and soybean, Positive	[48]
*GmDREB3*	*DREB* gene	Arabidopsis, Positive	[46,47]
*GmNEK1*	NIMA-related kinase gene	Arabidopsis, Positive	[53]
*GmEIN3*	Ethylene insensitive gene	Soybean, Negative	[51]
*GmMYBJ1*	MYB transcription factor	Arabidopsis, Positive	[62]
*SCOF-1*	C2H2 zinc finger gene	Arabidopsis and tabacco, Positive	[50]
*GmZF1*	C2H2 zinc finger gene	Arabidopsis, Positive	[67]
*GmPYL21*	ABA receptor family gene	Soybean, Positive	[48]
*Glyma11g13220(GmVRN1-like)*	Vernalization pathway gene	Arabidopsis, Positive	[59]
*GmTCF1a*	RCC1 family gene	Arabidopsis, Positive	[68]
*GmWRKY21*	WRKY-type transcription factor	Arabidopsis, Positive	[63]
*GmbZIP44*, *GmbZIP62 and GmbZIP78*	bZIP-transcription factor	Arabidopsis, Positive	[64]
*Hsp 70*	Heat shock protein genes	Soybean, Positive	[72]

Several studies conducted on soybeans have shown that Circular (circRNA)- and MicroRNA (miRNA)-based gene regulation were also involved in coordinating soybean responses to cold stress in post-transcriptional regulation [6,73,74,75,76,77,78]. In cold-treated soybean plants, miR166u, miR171p, miR2111f, and miR169c may regulate different targets in mature nodules through mRNA degradation [6]. Under chilling stress, Xu et al. [74] identified 51 miRNAs differentially expressed between chilling stress and control conditions in vegetable soybeans and indicated a negative relationship between the miRNAs and their targets. The recent finding indicates that soybean circRNAs might encode proteins and be involved in regulating low-temperature responses [76]. Kuczyński et al. [75] have identified target genes of five cold response-associated miRNAs and uncovered the nature of the correlation between target genes and the miRNAs. Transcription factors involved in cold stress response, such as *GAMYB* and *TCP,* are identified as possible targets of miR319 in Arabidopsis [78]. In contrast, *SBP-F*, *NAC-F*, and *NFY-F* are target genes of miR156a, miR164a, and miR169e in vegetable soybeans, respectively [74]. However, low-temperature inducible genes discussed in this review such as *CBF*, *WRKY*, and *MYB*, were not yet reported to be detected in association with soybean circular and microRNA. Similarly, no report indicated the involvement of transcription factors like GAMYB and TCP in soybean cold stress response.

As indicated above, homolog regulators identified in the soybeans exhibited a typical function of cold responsiveness in transgenic Arabidopsis. Nevertheless, it remains to be determined whether the regulators interacted with CBF and whether they are involved in the DRE-mediated or ABRE-mediated pathways. Arabidopsis cold responsive regulators including ICE1/2, CAMATA1-5, CESTA, BZR1/BES1, CCA1/LHY, SIZ1, CRLK1/2, OST1, EBF1/2, BTF3s, PIF3/4/7, 14-3-3s, CRPK1, EIN3, PRRs, MYB15, MPK3/6, phyB, and HOS1 regulate *COR* gene expression in CBF-dependent manner. Some of them (ICE1/2, CAMATA1-5, CESTA, BZR1/BES1, CCA1/LHY, SIZ1, CRLK1/2, OST1, EBF1/2, and BTF3s) have positive effects, while other (PIF3/4/7, 14-3-3s, CRPK1, EIN3, PRRs, MYB15, MPK3/6, phyB, and HOS1) have negative effects on CBF expression [79]. *GmEIN3*, a homolog of Arabidopsis *EIN3*, was identified to be involved in soybean cold response in a CBF-dependent manner and negatively regulate *GmDREB1* expression in transgenic Arabidopsis. Moreover, Arabidopsis regulators such as SAG12, WRKY33, ERF5, CZF1, RAV1, CZF2, MYB73, ZAT10, HSFC1, NPR1, HSFA1, RCF1, STA1, HOS15, GCN5, and HD2C were involved in cold stress response through CBF-independent pathway [79]. Similarly, soybean regulators GmMYBJ1, GmZF1, GmTCF1a, and SCOF-1 were identified to regulate cold responses in transgenic Arabidopsis in a CBF-independent pathway (Figure 1). A comparison of regulators in soybeans and Arabidopsis showed that, although different chilling temperature responsive regulators in soybean have been reported to regulate cold response in transgenic Arabidopsis, only a few of them were studied in detail to show the exact mechanism. Even though it requires further dissection for a precise understanding, the function of soybean cold-responsive regulators and associated *COR* genes studied in Arabidopsis shed light on the molecular mechanism of the cold response in soybeans and other crops.

Recent advancements in omics have provided us with access to genomic, transcriptomic, proteomic, and metabolomic data that can shed light on soybean cold tolerance [80]. Accordingly, QTLs and candidate genes associated with cold tolerance were identified using genome-wide association analysis and linkage mapping. Key regulators (TFs and genes) like soybean *CBF/DREB1,* which is differentially expressed in response to cold stress, were detected by transcriptomic studies [48,81,82]. The soybean proteomic study suggested that glutathione S transferase, sucrose binding protein, and dehydrins upregulated, while proteins responsible for cell division and growth, transcription, protein synthesis, and storage metabolism downregulated during cold stress [83]. Metabolomics studies can provide insights into multiple tolerance mechanisms at metabolic levels under abiotic stress. A study by Xu et al. [74] showed that CO_2_ tends to compensate for low-temperature stress fully or partially without other abiotic stress. Utilizing the omics and non-omics data, an advanced framework in molecular biology was constructed for detecting cold tolerance genes [77]. Pathway enrichment and crosstalk network research from the aspects of module analysis underlying cold tolerance provides a new way to identify cold-responsive genes and uncover the molecular mechanism of soybean cold-tolerance.

## 5. Conclusions and Future Perspective

Using genetic and molecular approaches, a number of relevant genes have been identified and new information has been continually discovered to enrich the cold-responsive pathway in soybeans. Elucidation of the mechanism regulating these complex and interactive signal transduction pathways is an important goal in achieving a full understanding of cold acclimation in soybeans. We believe that the inability of soybeans to increase *COR* gene expression during cold stress could be a little information to conclude as soybeans are cold-intolerant given the evidence that soybeans are grown in the Beijicun (North Pole, N 53°) Village of Mohe, Heilongjiang Province China. Hence, more work is needed to unravel the molecular machinery in soybean cold response.

Even though several studies have been done on soybean cold-responsive genes, most of the experiments were conducted using Arabidopsis host, e.g., *GmWRKY21*, *GmbZIPs*, *GmTCF1a*, *GmVRN1*, *GmZF1*, *GmMYBJ1*, *GmNEK1*, *GmDREB3*, *GmDREB1s*, *SCOF-1,* and so on. Experiments could be replicated in soybeans to verify whether the results were the same for soybeans. Moreover, orthologous genes of the upstream and downstream regulators that have been extensively described in Arabidopsis, must be further explored in soybeans. Our review indicated the involvement of both CBF-dependent and CBF-independent signaling pathways in soybean cold signal transduction. It is possible to confirm that the CBF cold-responsive pathway is not the only pathway in soybeans. More interestingly, an increased expression of *GmPYL21* mediated by *GmDREB1* under cold stress and the possible activation of downstream ABRE-mediated gene expression may be an interesting point of intersection between the two signaling pathways in soybean signal transduction.

Originating from the temperate regions, the soybean planting area has been moved northward to high-latitude regions in recent decades in China. It will continue to expand to high-altitude regions in the southwest of China. Thus, new varieties with cold tolerance are needed to adapt to low temperatures and short frost-free periods in high-latitude and high-altitude regions. Under the circumstance of these regions, soybeans are characterized by photoperiod insensitivity, early flowering, and early maturity for completing the life cycle before frost occurs. *E1*, *E2 E3,* and *E4* loci, major contributors in flowering and maturity, in high-latitude regions and *Gm ELF3* in high-altitude and low-latitude regions, are important target loci for genetic improvement related to growth periods. In addition, *GmVRN1,* described in the current review, can also be utilized as a cold-responsive gene to improve flowering time in the specific cold region. QTLs and molecular markers discussed in this review can be used for marker-assisted selection for soybean cold-tolerance. Regulators of cold response can be determined as target sites and be introduced by transformation or by precise modifications into the genome through genome editing. To create cold tolerate soybean cultivars, breeders always pyramid multiple genes into one line. Since cold tolerance is a multigenic trait controlled by many regulatory factors, future research on molecular mechanisms and regulatory pathways will facilitate the design of cold-tolerant varieties.

## Data Availability

All of the data are provided in the manuscript.
